# A Case of Jarisch–Herxheimer Reaction After Ceftriaxone Initiation for Lyme Carditis in Postpartum Female

**DOI:** 10.1155/crdi/5599926

**Published:** 2026-02-24

**Authors:** Emily Taylor, Sunam Sujanani, Ammara Khan

**Affiliations:** ^1^ University of New England College of Osteopathic Medicine, Biddeford, Maine, USA; ^2^ Department of Hospital Medicine, Northern Light Eastern Maine Medical, Bangor, Maine, USA

## Abstract

Lyme carditis is a complication occurring in 1%–5% of Lyme disease infections. It can lead to life‐threatening cardiac conduction abnormalities. Treatment with antibiotics may trigger the Jarisch–Herxheimer reaction (JHR), an acute inflammatory response to spirochetal lysis. Here, we present a 32‐year‐old postpartum female with complete heart block and disseminated erythema migrans who developed JHR within hours of ceftriaxone initiation, requiring vasopressor support. Hemodynamic instability resolved within 24 h, with subsequent normalization of cardiac conduction within 72 h. This case highlights the importance of recognizing JHR, distinguishing it from allergic reactions and septic shock, and avoiding unnecessary antibiotic discontinuation.

## 1. Introduction

Lyme disease is a prevalent tick‐borne bacterial anthropozoonosis caused by the spirochete *Borrelia burgdorferi sensu lato* (Bbsl) and transmitted by the *Ixodes* tick. Early localized (Stage 1) disease most commonly presents with erythema migrans, occurring in about 75% of cases, and is often accompanied by nonspecific symptoms such as fever and arthralgias. Early disseminated (Stage 2) disease may involve hematogenous spread to joints, the nervous system, eyes, and heart, while late disseminated disease (Stage 3) occurs years later with chronic arthritis and neurological manifestations [1, 2]. Lyme carditis is a rare complication of early disseminated disease, reported in 1%–5% of patients diagnosed with Lyme disease [3]. Lyme carditis presents with varying degrees of atrioventricular (AV) block, which may progress rapidly but often resolves with antibiotic therapy. Symptoms of Lyme carditis are typically chest pain, palpitations, dizziness, and syncope [4].

A clinically significant but lesser‐known complication of treating spirochetal infections is the Jarisch–Herxheimer reaction (JHR), a clinical syndrome occurring within 12–24 h of antibiotic treatment. JHR is an acute, self‐limited inflammatory response typically manifesting as a systemic shock‐like state with fever, chills, rigors, nausea, vomiting, headache, hypotension, tachycardia, myalgias, and exacerbation of preexisting skin lesions [5].

Although JHR is more frequently reported in other spirochetal infections such as syphilis and relapsing fever (RF), it is less common in Lyme disease [6–8]. The exact mechanism of JHR is unclear but thought to involve a strong cytokine‐mediated immunologic response to the release of endotoxin‐like factors from damaged spirochetes [9]. Here, we describe a case of JHR after ceftriaxone initiation for Lyme carditis in a postpartum patient.

## 2. Case Report

A 32‐year‐old female, three months postpartum with no prior past medical history, presented to her primary care physician with two weeks of fever, arthralgias, pleuritic chest pain, dizziness, and palpitations. Physical examination revealed multiple nonpruritic, blanching, erythematous, circular rashes on her arms, legs, and buttocks consistent with disseminated erythema migrans. She reported working in her yard but denied observing tick bites.

**FIGURE 1:**
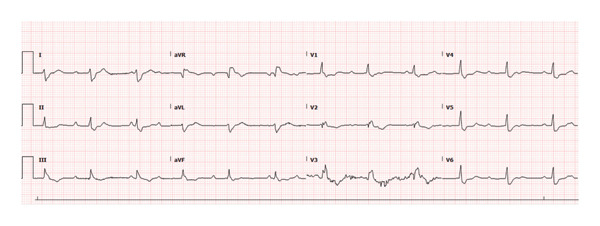
Complete heart block on ECG on hospital Day 1.

An electrocardiogram (ECG) performed at her primary care office showed complete heart block (CHB), prompting transfer to the emergency department (ED). Upon arrival, she was hemodynamically stable, and continuous cardiac monitoring revealed CHB with a stable underlying escape rhythm at the rate of 72 beats per minute (Figure 1). Lyme serology was obtained and returned positive for IgM and IgG antibodies.

She was diagnosed with Lyme carditis with CHB and started on intravenous (IV) ceftriaxone 2 g daily.

Three hours after the first dose of ceftriaxone, she developed hypotension with a blood pressure of 60/40 mmHg. Her heart rate remained unchanged between 70 and 80 beats per minute in CHB, with a respiratory rate of 19 breaths per minute, an oxygen saturation of 98% on room air, and a temperature of 36.9°C. She reported worsening myalgia, joint pain, and abdominal pain with associated nausea and lightheadedness. On exam, she was pale, diaphoretic, and had rigors with cool extremities. She also had an increased prominence in the appearance of her rash on her bilateral lower extremities. She had no urticaria, angioedema, or respiratory distress to suggest anaphylaxis.

She received a two‐L bolus of normal saline, which improved her blood pressure slightly to 79/52 mmHg. Although heart rate remained between 70 and 80 beats per minute, given the CHB and evidence of hypoperfusion, she was started on an IV dopamine drip titrated to a maximum dose of 6 mcg/kg/minute for presumed cardiogenic shock. This improved her blood pressure to 97/66 mmHg and her heart rate to 110 beats per minute. Laboratory studies at that time (Table 1) showed a transient leukocytosis with a white blood cell count of 18.2 × 10^9^/L. Lactate was mildly elevated at 1.6 mmol/L. Blood cultures were collected, which eventually returned negative. A stat echocardiogram revealed preserved biventricular systolic function, mild to moderate tricuspid regurgitation, and mild pulmonary hypertension. Of note, she also received IV ketorolac every 6 hours for joint pain.

**TABLE 1:** Laboratory values with reference ranges.

	Laboratory results	Reference ranges
Hemoglobin (Hg)	10.1 g/dL	12.0–16.0 g/dL
Hematocrit (Hct)	31%	36.0%–47.0%
White blood cell (WBC) count	18.2 × 10^9^/L	4.5–10.8 × 10^9^/L
Alanine aminotransferase (ALT)	ALT 54 U/L	0–33 U/L
Aspartate aminotransferase (AST)	AST 82 U/L	0–32 U/L
Alkaline phosphatase	18 U/L	35–104 U/L
Sodium	139 mEq/L	136–145 mEq/L
Potassium	3.7 mEq/L	3.5–5.0 mEq/L
Chloride	106 mEq/L	98–107 mEq/L
Urea nitrogen	13 mg/dL	6–20 mg/dL
Creatinine	0.57 mg/dL	0.40–1.10 mg/dL
Lactate	1.6 mmol/L	0.5–2.2 mmol/L
Erythrocyte Sedimentation Rate (ESR)	50 mm/hr	< 20 mm/hr
C‐Reactive Protein (CRP)	6.3 mg/L	< 3.0 mg/L

After 8 hours on a continuous IV dopamine drip, she was transitioned to norepinephrine drip titrated to a maximum dose of 0.06 mcg/kg/minute in an effort to maintain hemodynamic support while minimizing tachycardia. She was weaned off norepinephrine drip after 5 hours and remained hemodynamically stable after all vasopressors were discontinued. All of her symptoms had improved, and she was continued on IV ceftriaxone.

**FIGURE 2:**
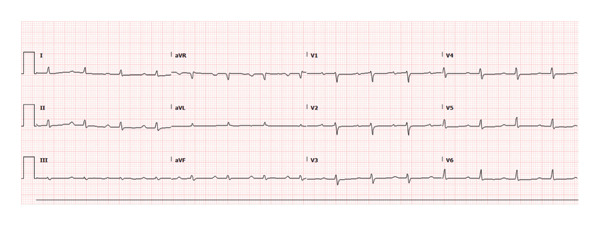
ECG from hospital Day 3 showing first‐degree AV block with PR interval 256 ms.

By hospital day three, telemetry monitoring showed resolution of CHB, with a transition to normal sinus rhythm with first‐degree AV block (Figure 2). Her leukocytosis had resolved (white blood cell count of 6.5 × 10^9^/L).

She continued to tolerate IV ceftriaxone without adverse effects. A follow‐up ECG obtained on hospital day four continued to show first‐degree AV block with a PR interval of 250 ms.

On hospital day five, she remained in first‐degree AV block. In light of clinical improvement and a PR interval below 300 ms, she was transitioned from IV ceftriaxone to oral doxycycline 100 mg twice daily for 21 days [3]. She was subsequently discharged with instructions to repeat the ECG with cardiology in 2 weeks. The patient was advised to avoid breast feeding for the duration of antibiotic therapy and for 5 days after the course ends due to teratogenic effects from doxycycline.

**FIGURE 3:**
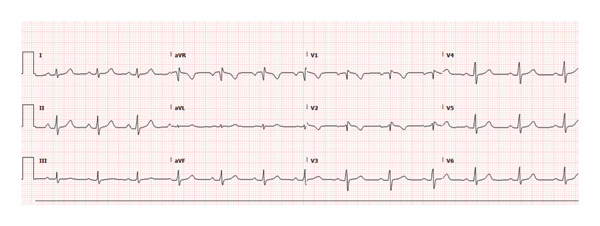
ECG from 2 weeks after discharge showing sinus rhythm with PR interval 178 ms.

At a 2‐week follow‐up after hospital discharge, the patient remained stable and asymptomatic. ECG demonstrated normal sinus rhythm and a PR interval of 178 ms (Figure 3). She continued to be monitored as an outpatient and had no further complaints.

## 3. Discussion

This case illustrates a classic presentation of disseminated Lyme disease with cardiac involvement. During this phase, Bbsl spirochetes can invade the myocardium, triggering an inflammatory response; notably, they have been isolated from the myocardium in such cases [10]. This disrupts the normal conduction pathways of the heart, leading to conduction abnormalities, most commonly AV block, which can range from first‐degree to CHB [3, 11].

Lyme carditis typically resolves with antibiotic treatment. However, hours after initiation of ceftriaxone, the patient exhibited a systemic reaction characterized by rigors, myalgia, and a transient worsening of her rash, which quickly progressed to a shock‐like state requiring fluid resuscitation and press or support.

The four main categories of shock—distributive (e.g., anaphylactic or septic), cardiogenic, hypovolemic, and obstructive—were considered in the differential diagnosis. Consideration was given to anaphylactic shock; however, it was deemed less likely given the absence of airway compromise, urticaria, pruritus, or eosinophilia. Furthermore, the reaction occurred several hours after the administration of ceftriaxone [5, 12]. Although not a classic presentation, anaphylaxis *can* occur within hours of exposure to antigens, however, the patient’s subsequent tolerance of ceftriaxone argues against an allergic reaction. Septic shock was also ruled out due to the rapid onset and resolution of the shock‐like state. Furthermore, Lyme disease does not cause septicemia, and a superimposed infection was ruled out due to negative blood cultures. Cardiogenic shock was ruled out based on echocardiographic findings showing preserved biventricular function and no signs of myocardial dysfunction. Hypovolemic shock was not supported by the clinical context, as there was no history of fluid loss or bleeding, and hemodynamic instability resolved without ongoing volume replacement. Obstructive shock was excluded given the absence of clinical or echocardiographic evidence of pulmonary embolism, cardiac tamponade, or tension pneumothorax. Recognition of JHR is critical to avoid unnecessary discontinuation of antibiotics or misdiagnosis as allergic reaction or septic shock.

The most common symptoms of JHR in spirochetal infections include fever (up to 90%), chills/rigors (up to 90%), headache (40%–80%), myalgia (40%–80%), and exacerbation of skin lesions (40%–80%) [13]. These percentages are best represented in syphilis and RF, where the reaction is more frequent and severe. In syphilis, the incidence of JHR is 8%–56%, while in tick‐borne RF, the incidence of JHR is reported as 54.1% [6, 7]. In Lyme disease, the overall incidence of JHR is reported between 7% and 30%, with similar but generally milder symptoms; however, specific percentages for each symptom in Lyme disease are not well defined in the literature [8].

This patient developed chills, rigors, headache, myalgia, and worsening of skin lesions shortly after antibiotic initiation. It is possible that the patient’s reaction including shock, was amplified by the presence of Lyme carditis, a higher spirochete load, and a reactive immune system likely due to the postpartum state [14]. Although fever is common in JHR, it is possible that therapy with IV ketorolac may have prevented fevers in this case. Given that other major categories of shock were excluded, the combination of this classic symptom profile, the reaction occurring 3 hours after the initiation of ceftriaxone, and the rapid clinical recovery made JHR the most likely diagnosis.

The JHR may be influenced by heightened immune reactivity, and the patient’s postpartum status possibly amplified her inflammatory response. It is known that the postpartum period is characterized by significant immunomodulation [15]. Pregnancy is associated with a shift toward an immunosuppressive, T helper cell (Th) 2‐dominant state to promote fetal tolerance. After delivery, the immune system undergoes a shift back to a Th1/Th17‐dominant, proinflammatory state which may increase susceptibility to exaggerated inflammatory responses [3, 16]. In the context of Lyme disease, this immune shift could intensify the patient’s response to spirochete lysis, resulting in a more severe JHR. Furthermore, Th1 cells secrete proinflammatory cytokines, including tumor necrosis factor α (TNF‐α), which has been implicated in the pathophysiology of JHR [9]. While this remains speculative, the interaction between postpartum immune rebound and severe JHR warrants further investigation. Fatal JHR has been documented in postpartum patients with other Borrelia infections, including a woman with tick‐borne RF who died within hours of antibiotic initiation shortly after delivery [17]. This supports the concept that postpartum immune rebound may contribute to exaggerated inflammatory responses following spirochete lysis.

Increased plasma concentrations of TNF‐α, interleukin‐6, and interleukin‐8 have been documented during JHR. One randomized controlled trial demonstrated that pretreatment with sheep anti‐TNF‐α suppressed the reaction following penicillin administration for louse‐borne RF, also reducing the associated proinflammatory cytokine surge [9, 18]. While speculative in the context of postpartum Lyme disease, these findings raise the possibility that cytokine dysregulation could contribute to severe JHR, and treatment with anti‐TNF‐α Fab may be promising in patients at higher risk.

In conclusion, this case highlights the importance of recognizing the JHR as a potential complication in the treatment of disseminated Lyme disease with cardiac involvement, particularly in vulnerable populations such as postpartum women.

## Funding

No funding was received for this manuscript.

## Conflicts of Interest

The authors declare no conflicts of interest.

## Data Availability

Data sharing is not applicable to this article as no datasets were generated or analyzed during the current study.
